# Come together: The importance of arts and cultural engagement within the Liverpool City Region throughout the COVID-19 lockdown periods

**DOI:** 10.3389/fpsyg.2022.1011771

**Published:** 2023-01-13

**Authors:** Melissa Chapple, Antonina Anisimovich, Joanne Worsley, Megan Watkins, Josie Billington, Ekaterina Balabanova

**Affiliations:** ^1^Department of Primary Care and Mental Health, University of Liverpool, Liverpool, United Kingdom; ^2^Department of English, University of Liverpool, Liverpool, United Kingdom; ^3^National Collaborating Centre for Mental Health, London, United Kingdom; ^4^Department of Communication and Media, University of Liverpool, Liverpool, United Kingdom

**Keywords:** arts engagement, mental health, wellbeing, online provision, COVID-19

## Abstract

**Introduction:**

Arts and cultural engagement activities have long been found to support wellbeing within the general population. In particular, community arts and cultural involvement during the COVID-19 pandemic have been an invaluable source of mental health and wellbeing support for many individuals across the globe. The initial move to remote engagement following the first United Kingdom lockdown demonstrated the importance of hybrid provisions, with isolated and vulnerable individuals finding online provisions important for wellbeing. With restrictions on movement and service access in the United Kingdom having gradually eased from March 2021, it is now important to explore how individuals navigated the ability to engage with either remote or in-person provisions. The current study aimed to explore the impact of the COVID-19 pandemic on arts and cultural engagement during periods of restrictions and initial easings on movement within the Liverpool City Region.

**Method:**

The study consisted of two waves of qualitative interviews within a broader longitudinal study. Twelve interviews were conducted during wave 1, which aimed to capture data during the initial COVID-19 lockdown period and the initial easing of restrictions. Eight of these participants were interviewed again for wave 2, which aimed to capture data during the winter 2020 lockdown period.

**Results:**

Framework analysis revealed three overarching themes: (1) The Importance of Arts and Culture for Personal Enrichment, (2) Belongingness through Socialization, and (3) Transitioning and Adjusting Throughout the COVID-19 Pandemic.

**Discussion:**

Findings presented in the current study provide further evidence of the value of arts and cultural activities in supporting wellbeing. Specifically, the current data emphasize the value of arts and cultural engagement throughout the COVID-19 pandemic and particularly during times of national restriction. Furthermore, the current study demonstrated that remote engagement provided important wellbeing support throughout the pandemic in a way that protected against mental health consequences, but with limitations on feelings of social connectedness within online environments. Amidst continuing risks from the COVID-19 virus and feelings of uncertainty, this study highlights the importance of hybrid provisions.

## 1. Introduction

Recent research has highlighted the short-term detrimental impacts that the COVID-19 pandemic and associated United Kingdom lockdown periods have had on the mental health and wellbeing of the British adult population (Iob et al., 2020; Pierce et al., 2020; Kromydas et al., 2021; Niedzwiedz et al., 2021; Wetherall et al., 2022). Specifically, findings have drawn attention to the mental health risks faced by vulnerable groups in particular, with increased mental health risks among individuals with pre-existing mental health conditions (Iob et al., 2020; Burton et al., 2021; Di Gessa et al., 2021; O’Connor et al., 2021; Wetherall et al., 2022); young adults (Niedzwiedz et al., 2021; O'Connor et al., 2021; Wetherall et al., 2022); women (Iob et al., 2020; Niedzwiedz et al., 2021; O’Connor et al., 2021; Wetherall et al., 2022); and individuals from racialized minorities (Iob et al., 2020; Katikireddi et al., 2021; Niedzwiedz et al., 2021). These mental health risks further interact with bigger physiological risks, with vulnerable groups also at an increased risk of developing long COVID symptoms following a COVID-19 infection (Thompson et al., 2021) and experiencing general healthcare disruption (Di Gessa et al., 2021). While individuals who were already experiencing mental health difficulties have been among the most vulnerable, findings have also highlighted mental health risks among the general United K population (Pierce et al., 2020). Specifically, around 29% of United Kingdom adults were found to have developed a common mental health condition (e.g., anxiety or depression) by April 2020, having reported no mental health conditions in the year before the COVID-19 pandemic (Chandola et al., 2020). However, the nation-wide scale of the United Kingdom lockdown periods and the impact of the broader COVID-19 pandemic mean that even individuals who have not developed a specific mental health difficulty have had to navigate fluctuating wellbeing (Robinson et al., 2022; Wetherall et al., 2022). Furthermore, mental health and wellbeing consequences have lasted beyond the initial lockdown period, with findings of decreased wellbeing during the initial easing of lockdown restrictions from July 2020 (Robinson et al., 2022; Wetherall et al., 2022) and worsened depression and loneliness from October 2020, alongside the return of lockdown restrictions in the United Kingdom (Wetherall et al., 2022). With ongoing risks to health posed by the COVID-19 virus and changing safety advice from the United Kingdom government, the fluctuating disruption to everyday life and individual wellbeing are likely to continue.

This rise in mental health struggles increases the demand for readily accessible services that can provide mental health support and encourage general wellbeing (Worsley et al., 2022). Healthcare services providing such support have faced pressures from before the COVID-19 pandemic that have resulted in reduced capacity to support patients (Allison et al., 2018; Cummins, 2018). These pressures together with the reduction in access to healthcare for mental health support during the pandemic highlight the need for research to focus on alternative methods of broader wellbeing support (Howarth et al., 2020; Younan et al., 2020; Worsley et al., 2022). In particular, community arts and cultural involvement during the COVID-19 pandemic has been an invaluable source of wellbeing support for many individuals across the globe (Mak et al., 2021; Meyrick and Barnett, 2021; Bu et al., 2022; Worsley et al., 2022). Importantly, research has shown that individuals in vulnerable groups and thus at risk of greater mental health consequences were often more likely to increase their arts and cultural engagement during the COVID-19 lockdown periods (Mak et al., 2021). However, findings also indicate that individuals from lower income families have reduced their engagement in arts and culture during the pandemic (Bu et al., 2022). This highlights a need to explore how restricted access to community support such as local arts and cultural activities has impacted on vulnerable individuals who may have struggled to access remote activities. Furthermore, Green et al. (2021) found that in-person, but not remote, contact was important in reducing depression and loneliness. By contrast, the findings show that weekly remote social engagement had little impact on individuals facing inequalities and struggling with symptoms of depression and loneliness. However, the initial move to remote engagement following the first United Kingdom lockdown demonstrated the importance of hybrid provisions, with isolated and vulnerable individuals finding online provisions important for wellbeing (Worsley et al., 2022). With restrictions on movement and service access in the United Kingdom having gradually eased from March 2021, it is now important to explore how individuals navigated the ability to engage with either remote or in-person provisions.

The current study aimed to examine the impact of the COVID-19 related reduced access to arts and culture engagement on wider wellbeing for individuals within the Liverpool City Region (LCR) at two critical time points: (Wave 1) during the October 2020 to February 2021 lockdown and (Wave 2) following the initial easing of restrictions from March 2021. For this exploration, the focus was on talking to individuals who had, to any extent previously engaged with arts and cultural activities before the COVID-19 United Kingdom lockdown periods. The aim of Wave 1 was to explore (i) the personal impacts of the lockdown periods starting March 2020 on access to arts and culture for individuals living within the LCR and (ii) any changes in how participants accessed arts and culture during the short-term easing of access restrictions throughout summer 2020. The aim of Wave 2 was to explore how the gradual easing of restrictions impacted on the ways and types of arts and cultural engagement for individuals within the LCR. This paper combines findings from Waves 1 and 2 to explore the fluctuations in arts and cultural access for individuals living within the LCR, and to further explore what these changes mean for future arts and cultural access beyond the COVID-19 pandemic.

## 2. Materials and methods

### 2.1. Participants

Participants were recruited *via* social media advertisement and through partner organizations’ publicity and communication channels (newsletter, webpages, and social media) into a larger longitudinal study which sought to explore the value of arts and cultural involvement throughout the COVID-19 pandemic in the LCR. A total of 12 participants (2 male, 10 female) took part in interviews conducted at wave 1 (January to February 2021), with eight (1 male, 7 female) of these participants also contributing data to Wave 2 (April–May 2021). Eligibility criteria required participants to be 18 years of age or over and fluent English speakers. Most of the participants (9 in Wave 1 and 5 in Wave 2) were aged 45 years or older. The participants were predominantly White British (11 in Wave 1 and 7 in Wave 2). Eight participants in Wave 1 and six participants in Wave 2 had used mental health services in the past or were using them at the time of participation. Four participants in Wave 1 and two participants in Wave 2 disclosed having a disability or multiple disabilities. The study was approved by the University of Liverpool Research Ethics Committee (reference: 7994).

### 2.2. Measures

#### 2.2.1. Interview schedule

Semi-structured interview schedules were devised for Waves 1 and 2 separately. For Wave 1 (see Supplementary material), structured questions focused on: (i) experiences of arts and culture pre-pandemic (e.g., motivations for engagement and benefits of engagement); (ii) engagement during the initial United Kingdom lockdown period in March 2020 (e.g., motivations to continue engagement, what was missed most and methods of access); (iii) engagement during lockdown easing during summer 2020 (e.g., how engagement changed during this time) and (iv) engagement during the winter lockdown (beginning December 2020 for the general United Kingdom population, but starting in October 2020 for many districts within the LCR due to local risk) and plans for future engagement (e.g., return, or not, to in-person engagement).

For Wave 2 (see Supplementary material), structured questions focused on: (i) overall sense of the impact of lockdown on arts and cultural engagement (e.g., were any activities missed) and (ii) plans to return to arts and cultural activities as restrictions continued to ease (e.g., plans to return to in-person activities and how participants were seeking out and evaluating whether to attend events).

### 2.3. Procedure

Participants took part in semi-structured qualitative interviews following informed written consent for each wave. For Wave 1, data were intended to capture: (i) the impact of the first United Kingdom lockdown (beginning March 2020) on arts and cultural engagement within the LCR; (ii) the impact of the short-term easing of restrictions from June 2020 and any changes to local arts and cultural engagement during this time; (iii) the impact of the winter 2020 lockdown (beginning December 2020) for the wider United Kingdom population on engagement and future plans to engage following further easing of United Kingdom restrictions throughout 2021. The 4th author, a female post-doctoral researcher who is trained in qualitative interviewing to doctoral level, conducted Wave 1 interviews.

For Wave 2, data were intended to explore: (i) the overall impact of the previous lockdown experiences upon engagement with arts and cultural activities within the LCR and (ii) immediate and future plans for arts and cultural engagement with restrictions on access to arts and culture easing within the United Kingdom. The 2nd author, who is also a female post-doctoral researcher trained in qualitative interviews to doctoral level, conducted Wave 2 interviews (upon handover from 4th author who started new role ahead of data collection for this wave).

All interviews across both waves were either conducted as video calls or telephone interviews due to COVID-19 restrictions. Interview duration ranged from 25 to 50 min.

### 2.4. Analysis

Audio data from interviews were recorded and transcribed verbatim. Transcription for both waves was completed by the first author who has prior experience of interview transcription for post-graduate research. Resultant transcripts were not returned to participants, as there were no areas of unclarity or missing data. Qualitative data from participant interviews were analyzed using Framework Analysis (Ritchie and Spencer, 1994). Framework Analysis was chosen due to its ability to incorporate inductive and deductive coding, preventing data loss while enabling comparisons across time points, a factor important for the larger study. The analysis was conducted within NVivo 10^1^ (Castleberry, 2014). Waves 1 and 2 were initially analyzed separately, with the final results combined due to large overlaps in data across the waves. The analysis stages were as follows:

1. Immersion: The first author transcribed interview data, keeping memos on points of interest to summarize the data to the rest of the team and for reference in later stages of analysis. For Wave 1, the 4th author made detailed memos on emerging ideas for each interview. For Wave 2, similar memos were made by the 2nd author. For both Waves 1 and 2, the memos created by the interviewing researchers consisted of field notes recorded at the time of interview as well as post-interview reflections.
2. Organizing: The first author sorted all data across Waves 1 and 2 into an organizational framework within NVivo 10. For Wave 1, the organizational framework was devised from the memo notes of key emerging ideas made by the 4th author in stage 1. For Wave 2, the final framework from Wave 1 was used to organize Wave 2 data in order to explore key differences and similarities between the two waves.
3. Indexing: The first author applied line-by-line coding to all Wave 1 and 2 transcripts within separate NVivo 10 files. For Wave 1 data, the first author selected three of the 12 transcripts that were felt to best capture the data. These three transcripts were then line-by-line coded by the 2nd and 3rd authors. These authors met with the first author to discuss resultant coding. Discussions established consistency and agreement in coding. Where codes differed between coders, agreement was achieved through further discussion of the data. For Wave 2, the 2nd author selected the three key transcripts, due to her familiarity with the original interviews. The same three coders discussed codes for Wave 2 and agreement was reached too. For both waves, a combination of inductive and deductive coding was used, relying on participants’ own language where possible (Saldaña, 2009).
4. Charting: The first author used reflections from stages 1–3 to recode data and move codes into initial subthemes and wider themes. Emerging themes were then discussed bi-weekly with the rest of the team and reframed to capture mutual understandings of the data.
5. Mapping: Initially, two frameworks were produced, one for Wave 1 data and one for Wave 2. This separation enabled the team to explore core similarities and differences between the two time points. Once both frameworks had been agreed upon by the whole team, it became evident that the findings from both waves were similar, and data from Wave 1 and 2 were combined.

## 3. Results

Three overarching themes were identified from the data: (1) The Importance of Arts and Culture for Personal Enrichment; (2) Belongingness through Socialization; (3) Transitioning and Adjusting throughout the COVID-19 Pandemic. A number of subthemes were identified for each overarching theme (see Table 1).

**Table 1:** Overarching themes and subthemes.

Themes	Subthemes
The importance of Arts and Culture for Personal Enrichment	Integration Within Personal Narratives and Identities
	Arts and Culture for Personal Growth
	Breaking from the norm
Belongingness through Socialization	Sharing Within Personal Networks
	Collective Feeling in Shared Spaces
	Local and Wider Community Involvement
Transitioning and Adjusting Throughout the COVID-19 Pandemic	Considerations Around Remote Inclusion
	Broadened Horizons
	Returning to In-Person Events

### 3.1. The importance of arts and culture for personal enrichment

#### 3.1.1. Integration within personal narratives and identities

All participants felt that engagement with arts and culture over time had played an important part in their lives. Many participants further emphasized the ways in which arts and culture had played a pivotal role in their lives, building on their sense of personal narrative and identity:

*[P1, W1] ‘Music’s sort of was my sort of thing, it’s what informed my working life and it’s also something I’ve been involved in with education and it’s something that’s always been part of my family, and I support the Phil[harmonic]‘s education programme.’*

*[P3, W1] ‘it’s been a big part of my work, but also my identity as well.’*

These values meant that access to arts and culture was of particular personal importance to all participants, especially in maintaining a sense of general wellbeing and positive mental health across time. Therefore, the restricted access to arts and culture during the COVID-19 lockdown periods had resulted in a sense of loss among participants:

*[P10, W1] ‘I’ve just felt sort of half the person I am because of this whole sort of area of me where I’m not finding any expression and just seems to have gone quiet.’*

*[P9, W1] ‘it’s almost like culture, which was always a large part of my life, when we all rushed home last March, I feel like I lost it on the way home’*

As illustrated by [P10, W1], participants who had felt arts and culture were an important cornerstone in their lives experienced a perceived loss of self.

For some participants, the lockdown restrictions had resulted in reduced engagement with arts and cultural activities, both personally and within community groups. This reduction was attributable to difficulties with translating activities online as well as broader difficulties that arose from living through the turbulence of the COVID-19 pandemic:

*[P9, W1] ‘I’ve really struggled to listen to music and to enjoy it the way that I did in the past…I’m finding it incredibly difficult to read more than a couple of pages at a time, I think I’ve spent too much time scrolling on my phone, just the same old pervasive boredom that most people have gone through really. I’ve not even accessed anything like Zoom gigs or anything like that. I feel like I dropped out really of all of it when lockdown happened’.*

*[P4, W2] ‘I stopped reading because I could not really concentrate on books. I stopped watching the television as well, other than odd things, because I could not find I could concentrate for long enough.*

However, there was a sense that the life-long value of arts and culture had led to resilience in the face of adversity, particularly for those who continued engaging remotely. This felt resilience helped during times of particular difficulty experienced within the COVID-19 pandemic:

*[P1, W1] ‘Yeah, I’m reaping the benefits now, years later, of things that happened to me in school, things that my family did…those things stayed with them, and they passed them on. So, I’m really, really pleased that I’ve had that experience that’s been there to fall back on.’*

*[P2, W1] ‘They [arts and cultural activities] are very much part of my life, and very much part of my mental health and wellbeing, there’s no doubt about that. I cannot imagine how I’d get here without having had the opportunity to do the distanced participation.’*

#### 3.1.2. Arts and culture for personal growth

Part of the value participants had found in arts and cultural activities over time stemmed from a sense of importance placed upon expanding the mind through creative exploration. Specifically, participants found value in the ability to learn new skills, take on new perspectives and build self-esteem:

*[P2, W1] ‘So, there’s been a sort of cross-traditional thinking that goes on, or used to go on, in the Everyman, which causes you to think through what our own attitudes are to modern times. We’re both in our seventies, so I think it’s important really, it keeps the mind turning over.’*

*[P9, W1] ‘It’s just being exposed to it, it’s what culture does is not it? It exposes you to different sounds, to different thoughts, to different experiences, to different visuals. It helps you grow, and keep adding more outlook I suppose, because you are being open to new ideas and new ways of expression all the time.’*

During the lockdown periods, this focus generally shifted toward continuing self-growth through remote engagement with arts and cultural activities. In particular, participants showed an interest in learning skills that enabled digital creativity and networking through the distribution of creative content *via* social networking services:

*[P1, W1] ‘I started trying to play with GarageBand (a software application to record, arrange and mix musics) …I managed to record three parts for the chorus and the first verse…it took me about a week really ‘cos I did not know what I was doing really but I was really pleased with myself’.*

*[P6, W1] ‘I started Instagram, I’d never used Instagram before last year, but I put the choir on Instagram and managed the Instagram account for the choir. You’re trying to get people, to raise the profile of the choir a bit, so I was following all sorts of people and reading things.’*

These explorations of remote engagement largely resulted in an increased willingness for creative risk-taking among participants. This moved them toward forms of arts and culture which they may not have otherwise accessed:

*[P10, W1] ‘my husband and I…probably take more risks going to things that we would not otherwise have done, online…I just think “oh, you know what, I’ll give that a try. I’ve got nothing better to do (laughs) so might as well give that a try.” I’ve probably gone to a broader range of things than I would have done pre pandemic, so that’s probably a good thing.’*

*[P12, W1] ‘it’s still a treat for me, time to get tickets to go and see live performances. And so, I am a little bit more conservative in what I choose and base it upon what I know I will like. So, because of the extent, it kind of stops you from taking a chance and experimenting a bit. But when it’s there for you, it allows you to say “oh, I may like this or may not it does not matter.” But I can try it out which is quite good.’*

In this way, the earlier mentioned sense of loss was balanced against a sense of opportunity among participants to broaden their horizons when it came to exploring arts and cultural activities that they could draw personal benefits from.

#### 3.1.3. Breaking from the norm

Before the pandemic, participants often engaged with local arts and cultural activities as a means to break up the tasks and strains associated with day-to-day life:

*[P12, W1] ‘my kind of day job is quite process driven, it’s really nice for me to participate in things [that] are just completely different and I find it just refreshes my mind, because it’s completely different. You know that saying about the change is as good as a rest, I find it really refreshing.’*

*[P8, W1] ‘You get engrossed in it and it’s a form of escapism, you are not thinking about all the other things going on like what you are having for tea, or what you need to pick up from the supermarket, you are just engrossed for that period of time.’*

It appeared that the use of arts and cultural opportunities in this way had led to the earlier mentioned integration of arts and cultural experiences within the personal narratives of the participants.

During times of national restriction, this sense of needing a break from the difficulties of everyday life became more apparent to participants. As a result, they actively tried to seek out enriching, often novel, experiences to break up and enrich their days:

*[P11, W1] ‘I tried to put some variety into the day. Maybe trying to do something different. Maybe trying to see it as an opportunity to try things that I would not normally try’.*

*[P2, W1] ‘I suppose belonging to the choir and having it to look forward to, and looking back on it after the rehearsal, looking back on what happened that day, in that rehearsal, and what we are supposed to get ready for next week, it sort of keeps me going.’*

In this sense, arts and cultural opportunities continued to offer similar, but now more important, benefits for participants during the lockdown restrictions as they had prior to the COVID-19 pandemic. However, at times where national restrictions had been eased, participants reported an increased desire to re-engage with arts and culture in person, as a means to fulfil this heightened need for escapism:

*[P4, W1] ‘when it was allowed. I thought ‘right I’m going down, and I’m going to go to some museums or exhibitions I have not seen.’ I got cheap tickets and a cheap hotel room and I just thought ‘just do it.”*

*[P2, W2] ‘the Trail of Light, I went to that. And I’ve seen some of the outside Biennial stuff when I have gone into town for a walk just to change the scenery.’*

Therefore, while remote opportunities to partake in arts and cultural activities were able to replicate some of the benefits of enriching day-to-day life, there was a sense that in person engagement offered a more rewarding opportunity for this purpose.

### 3.2. Belongingness through socialisation

#### 3.2.1. Sharing within personal networks

The personal gains that participants had experienced from engagement with arts and culture throughout life were found to enrich their social connections in similar ways. Before the pandemic, participants had relied upon arts and cultural activities as catalysts for forming deeper connections with close friends and family. These deeper connections emerged from imaginative creativity leading to more meaningful shared experiences:

*[PC, W1] ‘I enjoy going with people as well, it’s a part of who you are to share that experience with someone else…I think that’s become more and more clear that I love doing that, I love to be part of an experience with someone else’.*

*[P8, W1] ‘So, there’s the person that you are with, the fact that you are having that experience together, so you have that connection and then obviously after that you can always remember that experience and laugh or smile about it and tell the story.’*

Within the lockdown periods, there was a sense among participants that being able to access arts and culture within their social networks remained important for their wellbeing. As a result, participants had reported emulating these shared cultural experiences through online means. In this way, social media platforms and video call applications provided participants with an accessible way to re-engage with arts and culture within their personal social networks:

*[P3 W1] ‘My younger brother is interested in arts and culture as well, so we try, we are in two separate households but we sort of Facetime each other in the interval and things to reach out so it feels like we have gone to the theatre together a little bit [laughs].’*

*[P4, W1] ‘about four of my friends…we all started watching national theatre [laughs] online together on a Thursday night. So, we just made a point of Thursday national theatre online, and we’d all have a little WhatsApp group, and we would just pass comments.’*

However, most participants reported missing the depth of connection that was felt when engaging together in physical creative environments:

*[P10, W1] ‘for me, I most miss playing, being able to play with other musicians.’*

*[P6, W1] ‘I feel like I’ve gotten to know them quite well in book club over the years, because we have chatted once every month or six weeks. That is not the same on Zoom, it is not the same.’*

Overall, findings then indicate that online platforms (e.g., WhatsApp) enabled participants some sense of enriching social connection but in a way that could again not replicate the particular value of being together in-person for arts and cultural activities.

#### 3.2.2. Collective feeling in shared spaces

As well as enhancing personal social connections, these shared physical spaces were felt to enhance the shared experience among audiences. Specifically, the liveness that resulted in physical spaces created a sense of shared emotional contagion, enhancing both the personal and social benefits of the experience:

*[P3, W1] ‘I went to see the giants in Liverpool with my mum… we just caught the end bit, and we have got a video of us absolutely laughing and making the best time of it, it was just so magnificent, and I think in that moment everyone just felt this sense of like elation, and joy, and celebration’.*

*[P1, W1] ‘I just think that the feel of live theatre is brilliant because there’s a group of people there who in a way are exposing themselves in all sorts of ways and emotionally to you. And you are there with other people, and I find you experience emotions very intensely at the theatre.’*

Participants reported that these atmospheric benefits that had been felt in physical spaces were not replicable when connecting with others remotely. Instead, there was a sense of physical separation, which hindered the ability to feel moved together or personally within a shared experience:

*[P3, W1] ‘I’m kind of not getting is that shared experience. Me sitting at home with my headphones on does not give me the same butterflies in my belly or get me all angry and riled up about the world, in the same way that it might in a venue or when we are on the streets in a festival. That’s the thing I think, I feel I’ve not got from lockdown.’*

*[P8, W1] ‘say if it’s a concert, with the band themselves, when they are talking to the audience or they are singing a particular song, you sort of bounce off the reaction of the people on the stage as well as the people in the crowd. So, that goes then. So, it feels a little bit empty.’*

Additionally, participants had previously found value in the liminal spaces and surrounding social opportunities available during in-person engagements. In this way, physical spaces were found to personally and socially enrich arts and cultural events in a way that was lost when connecting remotely:

*[P3, W1] ‘you feel like you can go the bar afterwards and you can bump into someone and you are happy to have a chat about what you have just watched.’*

*[P5, W1] ‘You’d make a day or an evening of it. So, you’d go for a few drinks beforehand, you’d go to the event afterwards. So, it’s not just the participation, it’s what happens before and after.’*

Similarly, participants reported having previously felt a sense of emotional sharing with artists through engagement with their work or performances. However, this sense of connection was also hindered by lack of physical presence when engaging with arts online:

*[P6, W1] ‘it’s like the person who painted this was there too and you are not separated by the internet or television screen, because that’s the thing with being in the presence of something historical, you are actually there with something that’s hundreds or thousands of years old, seeing it on TV does not give you that same kind of emotional attachment’.*

#### 3.2.3. Local and wider community involvement

Among participants there was a strong sense of pride in belonging to the culture of the LCR due to the strong sense of community that was felt within the region, particularly in relation to the value of arts and culture that is felt within the city region:

*[P1, W1] ‘It’s been fab talking to people from other cities and from all over the world about how brilliant Liverpool is [laughs]…they are surprised sometimes that Liverpool has so much to offer in terms of so many different aspects of culture.’*

*[P12, W1] ‘I think one of the interesting things about the Liverpool City Region in particular is that you cannot even stand at the bus stop without having a chat with somebody. You catch somebody there and you start having a chat. And again, it’s another new perspective, so is like a community sort of experience.’*

During the lockdown periods, participants often felt unable to engage with this sense of local culture and community due to restricted access. Instead, they had a sense that the difficulties experienced throughout the pandemic transcended cultural identities, encouraging people to come together regardless of their geographical area. This shift toward a wider sense of community was enhanced by participants often finding it easier to engage with arts and cultural events outside of the LCR through remote provisions which were aimed at national over regional inclusion:

*[P12, W1] ‘It is nice though, because we are all in it together, moments where it does not matter kind of how wealthy you are, how intelligent you are, we are all on a level playing field.’*

*[P6, W1] ‘The National Theatre live stuff was fabulous but that’s obviously London based. I do not think I did anything else in Liverpool during the first lockdown.’*

However, as local arts and cultural activities began to move online, participants had expressed a keenness to re-engage with local services. Due to the sense of familiarity and identity evoked when engaging with local events remotely, it seemed that these opportunities were felt as more enriching:

*[P10, W1] ‘the Liverpool Phil(harmonic) then started doing their video on-demand concerts, some of those managed to have socially distanced audiences…I could deal with that ‘because they all played on that stage, I know a lot of people in the orchestra anyway quite well. I know the hall, I know what it’s like to sit there, so it did not feel as foreign’.*

*[P3, W1] ‘there’s usually a festival every May called LightNight …the guys moved the festival online so I kind of just sat on Facebook on that night and they’d release like a video of a choir doing a song or something like that, and that was quite lovely to feel a part of that.’*

This keenness to re-immerse in the LCR culture was particularly prominent during times when lockdown restrictions were eased and local provisions were once again accessible. During these times, participants were keen to re-visit local venues in person and engage with events that held a sense of personal and community relevance within the culture of the region:

*[P3, W2] ‘Linda McCartney had been married to Paul McCartney. And so, the photographs that she was taking was of a particular era and a particular kind of music and a particular kind of lifestyle…which obviously resonates with my own life memories of a certain period’.*

*[P4, W1] ‘I’d been to the Linda McCartney at the Walker and the Don McCullin at the Tate, I’d made a point of booking tickets for them.’*

However, participants expressed concerns for the future availability of such arts and cultural events within the LCR. In particular, they were worried about the impact of the pandemic on local artists and venues:

*[P9, W1] ‘How many venues, how many bands will still be going? How many theatre companies, how many theatres? How many art galleries? Are they going to be unable now to afford to put on the big hitting exhibitions? There’s so many unknown[s], even your favourite restaurant, is that still going to be there when all of this ends?’*

*[P10, W1] ‘An underlying concern now how the arts will continue… I do worry about very sheer variety of professional arts in Liverpool, how will that will be affected.’*

This concern appeared to particularly motivate participants further toward wanting to engage in local arts and cultural activities while they were available.

### 3.3. Transitioning and adjusting throughout the COVID-19 pandemic

#### 3.3.1. Considerations around remote inclusion

As highlighted in sections 3.1. and 3.2., the move from in-person to remote provisions resulted in a mixture of positive and negative responses from participants, with some aspects of arts and cultural engagement being seen as irreplaceable through remote provisions. Concerns raised among participants around the available remote provisions largely centered around the majority of engagement opportunities being delivered online. This frequently led to access barriers, with internet connectivity difficulties, inaccessible content and screen fatigue being common areas of difficulty for participants:

*[P6, W1] ‘We probably talk for about an hour and a half, maybe a couple of hours, about as much as I can cope with to be honest on the computer, I’ve had enough after about an hour and a half of looking at a screen.’*

*[P3, W1] ‘I’ve struggled to do workshops and if you are watching a piece of online theatre it freezes [laughs] and stuff like this, ah it is really irritating.’*

When barriers such as these arose during remote engagement, participants often chose to drop out of the activity altogether. For some, this hindered their motivation for wider engagement opportunities during the pandemic:

*[P1, W1] ‘I sort of missed the first one even though I was trying to connect and I just let it put me off. So, I did not bother with the rest of it.’*

*[P3, W1] ‘my internet was really bad and so I dropped out. So, that sort of put a stop to that.’*

Throughout the pandemic, some participants additionally experienced emotional difficulties in engaging remotely with arts and culture. These difficulties highlighted the changes and challenges brought about by the pandemic:

*[P10, W1] ‘they have this different version introducing the concert, and it was empty, there was nobody there… Then they did the concert, and I just wept, and wept.’*

*[P3, W1] ‘it was just really quite sad that they were like ‘so these are steps that you can do with a partner, but if you have not do it like this.’ And I was like feeling really… clearly making a point of me being on my own [laughs].’*

These difficulties largely related to the fact that participants were actively comparing remote engagement to in-person experiences. As a result, there was a sense that the format of many remote engagement opportunities offered a pale imitation of what had previously been available in person, leaving participants unfulfilled:

*[P8, W1] ‘I’m seeking connection and not getting that because it’s not interactive. So, it’s not quite filling the hole that I’m seeking it out to fill.’*

*[P6, W1] ‘I did try and do some of the Liverpool University concerts online, but I just could not, I do not know. I mean I watched them with my partner, but it just did not feel the same, slightly bizarre.’*

There was an indication as a result of these feelings that remote provisions should offer something that poses different benefits to in person events, as opposed to attempting to replicating in person events online.

#### 3.3.2. Broadened horizons

However, there was a sense that participants had begun to reframe the way they thought about remote provisions throughout the pandemic. As restrictions eased and the return to in-person events became more feasible, many had started to view remote engagement opportunities as complementary to the in-person ones, especially where it was felt that remote opportunities had suitably considered methods to enhance online engagement. In particular, participants were drawn to the improved accessibility that came with many remote engagement opportunities:

*[P3, W1] ‘my mum has a long-term health condition and sometimes going into environments can make that worse. And so, actually, to be able to access a place from home allows us to share that in a way that we either would not be able to before or it would have consequences which would cause stress or upset.’*

*[P11, W1] ‘if I was worried from a COVID point of view, or if I was ill, or I was unfortunate enough to have a long-term illness or disability, I’m really grateful that things have been made available in different ways.’*

Additionally, participants acknowledged that engagement with remote provisions provided an increased sense of control over events. In this way, they were better able to schedule arts and cultural activities into their lives, as well as to connect with new communities and cultures:

*[P3, W2] ‘Suddenly, the world as it were, become more readily available. You know, or I got more used to the fact that I can look at people who dance in different parts of Europe or Africa or Asia or South America, you know, it’s online and it’s just brilliant.’*

*[P8, W2] ‘One of the things that we did as part of that was, we collaborated on Zoom with a similar singing group in New York.’*

As a result of this reframing of remote cultural engagement, there was a sense among participants of wanting both online and in-person opportunities to continue after the pandemic:

*[P3, W1] ‘I do not think online activities will replace them necessarily, because I think talking to friends and things like that, I think everybody misses coming together with that sort of community feeling. But I think it will improve, I think it will be like an addition to [in-person], which is probably going to have quite wide-reaching benefits.’*

*[P5, W1] ‘I can see the benefit of both in a way ‘cos you think ‘I’ve got that live stream I can watch it again, like having a DVD.’ And then when they tour, or play, I’ll still go and see them anyway ‘cos that’s what people are like with bands’*

Some participants particularly appreciated how arts providers were finding new ways to maximize available services and methods of connecting people to arts and culture from home. In this way, remote opportunities became an interesting and unique way to connect with arts and culture:

*[P3, W1] ‘So, it was our job to kind of act as investigators…we would use Google maps to sort of help unravel everything…we’d all be talking on Zoom and then there would be someone in the chat function from the company who were hosting this event saying ‘I’ve got another clue for you.’ …they had really thought about how that platform benefited your experience. It wasn’t like, ‘just use Zoom and forget you are on Zoom.’ It was all integrated’.*

*[P5, W1] ‘on BBC radio four, where basically they wanted a different way of telling a story…they sent a postcard a day and each postcard had a bit of the story… you received several postcards, sent on different days over a two-week period. Then at the end of it you just literally read them, and they were significant to the story’*

As a result, there was a sense among participants that the value of remote engagement would remain beyond the COVID-19 pandemic if services continue to provide novel experiences. There was a sense that creative thinking was needed across society in order to explore how remote provisions might continue to offer experiences in addition to in person events, as opposed to seeking to replace them.

#### 3.3.3. Returning to in-person events

While many participants had indicated an interest to continue with remote provision, all participants were keen to return to in-person events. This was largely influenced by a desire to return to ‘normality’:

*[P7, W1] ‘As soon as I can get out and do the things I used to do I will be and with other people’.*

*[P9, W1] ‘I am very much looking forward to getting back into that rhythm of accessing culture regularly and being part of a community, I cannot wait for that. It’s getting me through.’*

However, participants were continuing to balance this desire for a return to life in person with the risks that COVID-19 posed in accessing in-person venues. Many participants felt that remote provisions would remain important during this transition period, despite a desire to re-engage in in-person events:

*[P11, W1] ‘there’s going to be a quite long period, I think of adjustment, rebalancing, considering risk. Say in a few months’ time if things started to reopen, would I want to go to them? Or would I still prefer online? I guess there’s going to be a transition period.’*

*[P2, W1] ‘it’s an art gallery so you are not really talking much at all to the other people, because you are going around looking at whatever you are looking at for yourself. But just the fact of other people being around, although they were all properly spaced, but you could see there were other people around.’*

A particular difficulty among participants during this transitional period was continued uncertainty; participants felt unable to plan ahead in regards to arts and cultural engagement, due to concerns that restrictions might be re-introduced:

*[P5, W1] ‘Well, it’s frustrating the sort of stop-start motion to all our lives, but I understand the reason.’*

*[P1, W2] ‘when we were still in lockdown, the roadmap has just been released, so I did not plan anything in advance when that started to happen. It’s always been just waiting until it has happened, and then waiting until everybody else would rush out and do stuff, and then see what I feel like doing.’*

While many participants had been planning to return to in-person events as soon as opportunities became available, the ongoing health risks of COVID-19 created an overall sense that participants wanted to see remote provisions continue. In particular, the data presented here highlighted the importance of hybrid participation options for in-person events as well as the inclusion of remote opportunities that sought to offer some new experience that drew on the online environment.

## 4. Discussion

### 4.1. Summary of findings

The current study aimed to explore the impact of the COVID-19 pandemic on arts and cultural engagement during periods of restrictions and initial easing on movement within the LCR. Wave 1 data were collected to explore the impact of the lockdown periods on involvement with arts and culture and any resulting benefits from engagement. Wave 2 data aimed to explore changes associated with the gradual easing on movement restrictions.

#### 4.1.1. Arts and culture supporting mental wellbeing

Findings presented in the current study provide further evidence of the value of arts and cultural activities in supporting wellbeing (Coulton et al., 2015; Fancourt et al., 2019; Fancourt and Finn, 2019; Fancourt and Steptoe, 2019; Fancourt and Tymoszuk, 2019; Worsley et al., 2022). Specifically, the current data emphasized the value of local arts and culture within personal narratives and personal identities. This intrinsic value of arts and culture led some participants to feel an initial loss of self with the imposing of lockdown restrictions that negatively impacted wellbeing. However, among those who continued to engage with arts and culture through online means, there was a sense of resilience in the face of adversity that promoted wellbeing. This supports previous findings that arts and cultural engagement during the COVID-19 pandemic has provided a source of mental health support (Mak et al., 2021; Meyrick and Barnett, 2021; Bu et al., 2022; Worsley et al., 2022).

As previously demonstrated by Worsley et al. (2022), participants highlighted the importance of regular remote engagement throughout the lockdown periods in giving them something to look forward to, a break away from the difficulties created by the pandemic. In general, participants felt that arts and culture had always been an important break away from the mundane aspects of day-to-day life, becoming more important during times of adversity. The value demonstrated through remote access indicates that the continuation of remote provision may prove a useful means of engagement for those unable to engage in-person beyond the pandemic. In particular, remote engagement allowed some form of social engagement, making participants feel more connected. These findings somewhat contradict previous findings that in-person but not remote contact was important for reducing depression and loneliness (Green et al., 2021). While there was a general sense that in-person social contact boosted wellbeing in ways that could not be identically replicated remotely, remote contact still promoted wellbeing for many participants.

#### 4.1.2. Comparing remote access to in-person engagement

The inability of remote engagement to completely replicate the enjoyment and associated benefits of in-person engagement was emphasized by participants. Specifically, online environments were unable to provide smaller but important social experiences around events and liminal spaces, such as the walk back from an event, which boost opportunities for personal reflection and social connecting. These results further develop findings that remote contact does not reduce loneliness in the same way as in-person engagement (Green et al., 2021). Together with findings on mental health and wellbeing support (see “Arts and culture supporting mental wellbeing”), the current study demonstrates that remote arts and cultural engagement can reduce feelings of loneliness but in a way that is perhaps not as socially enriching as in-person events. This left participants largely keen to return to in-person events to experience a sense of togetherness with artists and other attendees.

Furthermore, while remote engagement offered some benefits during the pandemic, many barriers to access were raised across participants. Specifically, online remote events were associated with screen fatigue and were often impeded by problems with internet connectivity. These barriers sometimes resulted in disengagement and raised concerns among the current sample for others in their communities who they felt were of lower social economic status and who may not have the same means and opportunities to access online events. Together with previous findings reporting reduced remote engagement among lower income families (Bu et al., 2022), this further highlights the need for remote activities to provide varying means of engagement that are accessible to all individuals within local communities.

#### 4.1.3. Potential for hybrid models

Despite the limitations of online engagement, many participants were keen for remote opportunities to continue beyond the pandemic. Upon the initial easing of movement restrictions, participants tended to reframe their view of remote opportunities from a pale imitation of in-person events to something that could potentially offer added value: in addition to rather than instead of. However, current remote provisions were perceived as not reaching their full potential to offer complimentary experiences. This arose from limitations of current software and online environments, which had tended to encourage the replication of in-person ways of connecting rather than encouraging new and interesting ways to connect together with arts and culture. This highlights a need for new and innovative means of remote engagement that can add value to in-person opportunities as well as introducing new or adapted ways to connect online. Specifically, current methods of audience engagement and wider social connection were limited and often had to be initiated through social media rather than being easily integrated within services. Such adaptations and potential developments to online environments could potentially enhance the mental health and wellbeing benefits offered through remote engagement (Bu et al., 2022).

In the immediate future, it was felt that it will be important for remote opportunities to continue and to further develop. Although restrictions in the United Kingdom have since lifted entirely at the time of writing, the perceived risks of the COVID-19 virus meant that some participants felt safer engaging online, with others returning to some in-person events but finding value in the ability to take part online to reduce potential COVID-19 exposure. Together with prior findings (Mak et al., 2021; Meyrick and Barnett, 2021; Bu et al., 2022; Worsley et al., 2022), this highlights a need for arts and cultural activities to continue online even with the return of in-person opportunities in order to offer an alternative means of mental health and wellbeing support for those who are unable to engage in person. Furthermore, participants found that remote engagement increased their connectivity with other communities, while in-person events allowed them to connect with their local culture. In this way, a hybrid of in-person and remote arts and cultural activities holds the potential to enhance the social and wellbeing benefits of arts and cultural involvement (Coulton et al., 2015; Fancourt et al., 2019; Fancourt and Finn, 2019; Fancourt and Steptoe, 2019; Fancourt and Tymoszuk, 2019; Worsley et al., 2022).

### 4.2. Limitations and future research

The majority of participants included in the present study engaged with remote arts and cultural activities *via* online provisions. Therefore, the sample is not representative of individuals who have not had access to an internet connection during the COVID-19 pandemic. Therefore, it is important for future research to explore arts and cultural access throughout the pandemic among low socio-economic status households, particularly those who have had some level of reduced access to online resources for remote engagement. Similarly, the current study did not focus specifically on demographics who may face particular social disadvantages such as individuals from racialized minorities, individuals with pre-existing mental health conditions, young adults and women (Bu et al., 2022). This is particularly important due to the mental health risks faced by such individuals who are socially disadvantaged (Bu et al., 2022). Therefore, research which takes a particular focus on these groups is necessary to inform ways in which hybrid provisions can be adapted in order to remain inclusive, particularly among those in need of community wellbeing support. Furthermore, future research should explore differences in engagement among those who had been experiencing pre-existing mental health conditions due to the increased mental health risks faced by this group during the pandemic (Iob et al., 2020; Burton et al., 2021; Di Gessa et al., 2021; O’Connor et al., 2021; Wetherall et al., 2022).

## 5. Conclusion

In conclusion, the findings of the present study build upon previous research (Mak et al., 2021; Meyrick and Barnett, 2021; Bu et al., 2022; Worsley et al., 2022) to emphasize the value of arts and cultural engagement throughout the COVID-19 pandemic and particularly during times of national restriction. Furthermore, the current study provides further insight into the differences between remote and in-person engagement in resulting wellbeing (Bu et al., 2022). Here it was demonstrated that remote engagement provided important mental health support throughout the pandemic, but with limitations on feelings of social connectedness within online environments. Amidst continuing risks from the COVID-19 virus and feelings of uncertainty, this study highlights the importance of hybrid provisions. However, further research is needed to explore how in-person and remote engagement has continued to change with the full easing of restrictions on access.

## Data availability statement

The raw data supporting the conclusions of this article will be made available by the authors, without undue reservation.

## Ethics statement

The studies involving human participants were reviewed and approved by University of Liverpool Research Ethics Committee (reference: 7994). The patients/participants provided their written informed consent to participate in this study.

## Author contributions

JB and EB conceived the study. MW and AA collected the qualitative data. MW and AA coded a subset of transcripts. MW identified emerging themes from Wave 1 data and produced preliminary findings which were presented at a partner meeting. MC analyzed the qualitative data to finalize the framework, in consultation with the research team. MC wrote the first draft of the manuscript. JB, EB, JW, AA, and MW read, commented on, and revised the manuscript providing important intellectual input. All authors contributed to the article and approved the submitted version.

## Funding

The project was funded by the Arts and Humanities Research Council (AHRC) as part of UK Research & Innovation COVID-19 funding.

## Conflict of interest

The authors declare that the research was conducted in the absence of any commercial or financial relationships that could be construed as a potential conflict of interest.

## Publisher’s note

All claims expressed in this article are solely those of the authors and do not necessarily represent those of their affiliated organizations, or those of the publisher, the editors and the reviewers. Any product that may be evaluated in this article, or claim that may be made by its manufacturer, is not guaranteed or endorsed by the publisher.

## Footnotes

^1^An older version of NVivo was used due to a limited access to newer versions during the Covid-19 pandemic.
