# Supplementary material for: Come together: The importance of arts and cultural engagement within the Liverpool City Region throughout the COVID-19 lockdown periods
Source: Front Psychol. 2023 Jan 13;13:1011771. doi: 10.3389/fpsyg.2022.1011771 (PMC9880199; doi:10.3389/fpsyg.2022.1011771)
Supplement: Supplementary file 1 [file Data_Sheet_1.DOCX]

Supplementary Material

# 1. (Qualitative) Interview Schedule (Wave 1)

1. **Welcome and Introduction (5 mins approx.)**
2. **Experiences prior to lockdown (5-10 mins approx.)**
3. Can you describe your experience of arts and cultural activity in the Liverpool City Region before the COVID-19 lockdown period?
4. What motivated you to participate in arts and cultural activity in the Liverpool City Region before the COVID-19 lockdown period?
5. What do you enjoy about engaging in arts and cultural activity? What benefits would you say you derive from it?
6. **Impact of Lockdown (10-15 mins approx.)**
7. How did the COVID-19 lockdown period have an impact upon your engagement with arts and cultural activity in the Liverpool City Region?
8. Which kind of arts and cultural activity in the Liverpool City Region did you engage in during the COVID-19 lockdown period?
9. What motivated you to participate in arts and cultural activity in the Liverpool City Region during the COVID-19 lockdown period?
10. Which arts and cultural activities did you most enjoy and why?
11. Which arts and cultural activities did you most miss and why?
12. How did you get access to arts and cultural activity in the Liverpool City Region during the COVID-19 lockdown period?
13. Did you find some activities easier/more difficult to access than others? If so, which ones?
14. **Resulting Changes (5-10 mins approx.)**
15. How has your experience of arts and cultural activity in the Liverpool City Regions changed since the easing of COVID-19 lockdown? (How far have you resumed former activities? Or continued with those taken up during lockdown?)
16. Is there anything else you would like to say?
17. **Thank you and debrief (5 mins approx.)**

# 2. (Qualitative) Interview Schedule (Wave 2)

1. **Welcome and Introduction (5 mins approx.)**
2. **Arts and Culture engagement (10-15 mins approx.) Thinking about the last two months.**
3. How did the COVID-19 lockdown period have an impact upon your current engagement with arts and cultural activity in the Liverpool City Region?
4. Which kind of arts and cultural activity in the Liverpool City Region did you engage in?
5. What motivated you to participate in arts and cultural activity in the Liverpool City Region?
6. Which arts and cultural activities did you most enjoy and why?
7. Which arts and cultural activities did you most miss and why?
8. How did you get access to arts and cultural activity in the Liverpool City Region during the last two months?
9. Did you find some activities easier/more difficult to access than others? If so, which ones?
10. **Changing experiences (5-10 mins approx.) Thinking about the most recent easing of lockdown (Spring 2021).**
11. How has your experience of arts and cultural activity in the Liverpool City Regions changed since the easing of COVID-19 lockdown? (How far have you resumed former activities? Or continued with those taken up during lockdown?)
12. What are your plans and thoughts on arts and cultural activity after easing of lockdown?
13. Is there anything else you would like to say?
14. **Thank you and debrief (5 mins approx.)**
